# Application of Layered Strain Technique in NSTE-ACS

**DOI:** 10.1155/2022/2426178

**Published:** 2022-01-18

**Authors:** Nan Zhao, Luyao Zhang, Xijun Zhang, Chuang Li, Yang Li, Peng Qian, Yu Lu

**Affiliations:** ^1^Department of Ultrasound, Henan Provincial People's Hospital, China; ^2^Department of Anesthesiology and Perioperative Medicine, Henan Provincial People's Hospital, China; ^3^Department of Geriatrics, Henan Provincial People's Hospital, China

## Abstract

**Background:**

To explore the application value of layered strain technique in non-ST elevation acute coronary syndrome (NSTE-ACS).

**Methods:**

120 patients with suspected NSTE-ACS undergoing coronary angiography in our hospital from December 2018 to December 2019 were prospectively selected. According to the results of coronary angiography, the patients were divided into the significant CAD group and the nonsignificant CAD group. Echocardiography was performed 1-2 hours before invasive coronary angiography. The long axis and circumferential strain of the endocardium, myocardial layer, and epicardium were evaluated by the layered strain technique. The territorial longitudinal strain (TLS), the global longitudinal strain (GLS) of the three myocardial layers, and the global circumferential strain (GCS) were calculated based on the perfusion region of the three coronary arteries and the 17-segment model of the left ventricle. The primary endopoints were TLS and GCS of the three-layer myocardium.

**Results:**

Compared with the nonsignificant CAD patients, the TLS and GCS of three-layer myocardium in significant CAD patients were decreased, especially in the endocardium. The absolute values of TLS and GCS of the endocardium and epicardium in significant CAD patients were lower than those in nonsignificant CAD patients. This indicates a significant decrease in endocardial function. Receiver operating characteristic (ROC) curve analysis showed that endocardial TLS was superior to LVEF, Troponin I (TnI), and other strain parameters in evaluating the extent of coronary lesions.

**Conclusions:**

The layered strain technique of 2D-STE can evaluate the severity of coronary lesions in patients with NSTE-ACS, and for significant CAD patients, endocardial function is significantly more impaired than epicardial function.

## 1. Background

Acute coronary syndromes (ACS) include non-ST elevation acute coronary syndrome (NSTE-ACS) and ST elevation myocardial infarction (STEMI). Coronary artery obstruction in STEMI requires urgent revascularization, but the clinical presentation of patients with NSTE-ACS is much more variable, and about one-third of patients with NSTE-ACS do not necessarily have coronary artery obstruction or severe coronary lesions, and these patients do not require revascularization. Therefore, it is more important for clinicians to accurately select patients who really need coronary angiography for revascularization to reduce the complications related with the operation and also reduce the medical costs during the treatment process [1]. Two-dimensional speckle tracking echocardiography (2D-STE) allows quantitative evaluation of the myocardial strain to assess cardiac function and has been considered an accurate indicator of overall and local cardiac function [2]. Compared with conventional echocardiography, strain ultrasound can be more sensitive and accurate in identifying the degree of coronary lesions in patients with NSTE-ACS [3]. The latest layered strain analysis software can evaluate the myocardial morphology of the endocardium, myocardial layer, and epicardium separately, among which the endocardium is the most sensitive to myocardial ischemia, and careful evaluation of the endocardial myocardium can improve the diagnostic accuracy of CAD [4]. In this study, we applied the layered strain technique to evaluate the myocardial morphology of three layers in patients with suspected NSTE-ACS.

## 2. Methods

### 2.1. Study Objects

120 patients with suspected NSTE-ACS admitted to our hospital from December 2018 to December 2019 were prospectively collected. Suspected NSTE-ACS was diagnosed by chest pain, ECG changes, troponin values etc. Electrocardiography was completed on admission, and electrocardiographic ST depression and T-wave changes suggested myocardial ischemia. Echocardiography was completed within 1-2 hours before coronary angiography or within 48 hours of the onset of chest pain, and the echocardiographic data and clinical data were done double-blind. Exclusion criteria were as follows: severe arrhythmias, severe valvular lesions, history of myocardial infarction, atrial fibrillation, myocarditis (diagnosed by clinical symptom, myocardial enzyme, etc.), left bundle branch block, and poor image quality. The primary endopoints were TLS and GCS of the three-layer myocardium; this is the difference between endocardial and epicardial strains measured with the echocardiographic technique. All patients signed an informed consent form and received medication according to the latest guidelines, and this study was approved by our medical and health research ethics committee.

### 2.2. Instruments and Methods

#### 2.2.1. Echocardiography

A GE vivid E9 echocardiograph with an M5S probe and the frame rate ≥ 55 beats/min was used. The left ventricular end-diastolic volume and LVEF were evaluated by the biplane Simpson method. 2-dimensional gray-scale images of three consecutive cardiac cycles were acquired at end expiration, including apical 4-chamber, 2-chamber, and 3-chamber views and standard short-axis views at the level of the parasternal papillary muscle. The long-axis strains of the endocardium, myocardial layer, and epicardium were recorded in the apical views, and the circumferential strains were recorded in the parasternal short-axis views. The longitudinal and circumferential strains of 16 longitudinal segments and 6 circumferential segments were analyzed, and the global longitudinal strain (GLS) and global circumferential strain (GCS) were obtained by averaging all segments of each myocardial layer. Investigators with poor segmental tracking could manually adjust, and if tracking was consistently poor, the segment was excluded from the study. Territorial longitudinal strain (TLS) was calculated by averaging the perfusion areas of each of the 3 major coronary arteries in the 16-segment model of the left ventricle.

#### 2.2.2. Coronary Angiography

Coronary angiography was performed in all patients, and each coronary stenosis was visualized by multisection projection to avoid side branch overlap and shortening of the associated coronary stenosis. Patients were divided into the significant CAD group and nonsignificant group according to the coronary angiography, and the significant CAD group was defined as at least one coronary lumen stenosis ≥ 50%.The nonsignificant CAD group was defined as no one coronary lumen stenosis ≥ 50%.

### 2.3. Statistical Analysis

The data are expressed as mean ± standard deviation, value (percentage), median (interquartile range). Independent *t*-test was used for continuous data of two groups, and chi-square test was used for the categorical data of two groups. The upper leftmost value on the ROC curve best reflected the sensitivity and specificity of endocardial, myocardial, epicardial TLS, GLS, GCS, TnI, and LVEF in predicting severe CAD, and the area under ROC (AUC) of each parameter was calculated. Logistic regression was applied to analyze the potential factors affecting cardiac function, such as age, BMI, systolic blood pressure, diastolic blood pressure, hypertension, diabetes mellitus, and each parameter of echocardiography; *P* < 0.05 was considered statistically significant.

## 3. Results

### 3.1. Comparison of Angiography

Angiography showed that 75 patients had significant CAD, and 45 patients had nonsignificant CAD. Among the patients with significant CAD, 21 patients had one coronary artery stenosis, 35 patients had two coronary arteries occlusion, and 19 patients had three coronary artery occlusions.

### 3.2. Comparison of General Data

There were no significant differences in age, gender, height, weight, body surface area, smoking history, hypertension, diabetes, blood lipid, systolic blood pressure, diastolic blood pressure, and heart rate between the two groups (*P* > 0.05) (see Table 1). In the CAD group, 59 cases were larger than 0.2 ng/mL and 16 cases were smaller than 0.2 ng/mL. In the non-CAD group, 26 cases were larger than 0.2 ng/mL and 19 cases were smaller than 0.2 ng/mL.

### 3.3. Echocardiography

In all patients, endocardial TLS, GLS, and GCS were numerically higher than epicardial TLS, GLS, and GCS (*P* < 0.001). The TLS, GLS, and GCS of the three-layer myocardium in patients with significant CAD were damaged to varying degrees compared with those in patients with nonsignificant CAD, but the endocardial TLS, GLS, and GCS had the most pronounced effect. The absolute difference of TLS, GLS, and GCS between the endocardium and epicardium in patients with significant CAD was lower than that in patients with nonsignificant CAD (*P* < 0.001). This indicates that the central endocardial function is significantly decreased in significant CAD patients. Compared with the global strain, the absolute difference of the endocardial and epicardial strains is generally lower in the local strain. However, the differences between these parameters are not statistically significant (see Tables 2 and 3).

**Figure 1:**
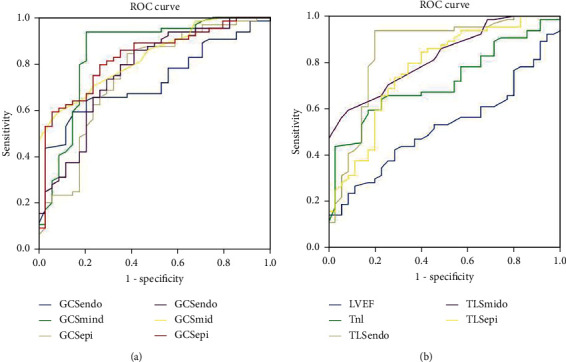
The ROC curve was used to evaluate the ability of LVEF, TnI, and strain parameters in diagnosing patients with significant CAD.

Taking the results of coronary angiography as the gold standard, the ROC curve was used to evaluate the ability of LVEF, TnI, and strain parameters in diagnosing patients with significant CAD. The results showed that the endocardial TLS and middle TLS were better than epicardial TLS, and the endocardial GLS and GCS were better than epicardial GLS and GCS. The area under the ROC curve of strain parameters was greater than LVEF and TnI (see Table 4 and Figure 1). Multivariate regression analysis showed that the decrease of endocardial TLS was the only predictor of significant CAD, with OR value of 2.15 and 95% CI of 1.45-3.10, which was not related to the variables included in the model besides endocardial TLS.

### 3.4. Repeatability Test

The intraobserver and interobserver consistency tests of each parameter are shown in Table 5. The data showed that the layered strain parameter had good repeatability.

## 4. Discussion

Approximately two-thirds of patients with NSTE-ACS require coronary angiography, and several different noninvasive methods to identify CAD are important in clinical work; ECG and troponin alone often fail to identify patients with obstructive CAD. Coronary CT angiography can improve diagnostic accuracy, but its clinical use is limited by being expensive or not available for emergency applications [5]. In this study, we introduced the assessment of left ventricular longitudinal and circumferential strain with 2D-STE as a new method to identify patients with suspected NSTE-ACS, showing impaired left ventricular function in all three myocardial layers in patients with significant CAD compared to patients with nonsignificant CAD.

The left ventricular wall is composed of three layers of myocardium, and the deformation and function of each myocardial ventricular wall layer varies during cardiac contraction. The endocardium thickens and shortens more during systole than the epicardium. Myocardial infarction models [6] and myocardial infarction reperfusion studies have shown that the endomyocardial layer is first affected by ischemia, and as the severity increases, ischemia and necrosis spread from the endocardium to the epicardium. The endocardium is most susceptible to ischemia, so assessing changes in the myocardial strain in the endocardial layer can predict CAD [7].

Traditionally, the whole myocardial wall thickness is considered in the evaluation of myocardial function, without considering the differences between the layers of the myocardium. The deformation of each myocardial layer is not independent, and contraction of the viable myocardium may lead to deformation of the adjacent nonviable myocardium by traction. Similarly, the nonviable myocardium may negatively affect the contraction of the adjacent viable myocardium. The deformation of each layer of myocardium is the sum of the active contraction within that layer and the passive traction of the adjacent myocardium. Therefore, layered strain analysis may improve diagnostic accuracy in these patients. The layered strain technique may increase the understanding of the morphology and pathophysiology of myocardial ischemia, and decreased endocardial function in patients with coronary artery disease can be caused by coronary artery obstruction, myocardial injury, and myocardial hibernation [8, 9]. In our study, endocardial longitudinal and circumferential strains were better than epicardial strains to identify patients with significant CAD.

### 4.1. Limitations of the Study

The definition of myocardial stratification is arbitrary and is simply divided into 3 parts. Only longitudinal and circumferential strains were evaluated in this study, excluding radial strains, which have methodological limitations and have been shown to be inferior to longitudinal and circumferential strains in identifying ischemia and necrosis [10]. Due to the poor reproducibility of strain measurements in the basal and apical segments, we selected only short-axis sections of the intermediate segments, and therefore, the results of the analysis were not comprehensive. We did not compare whether layered strain analysis has a more important value in the diagnosis of CAD compared to the conventional global strain analysis. Patients were not followed up, and it is not known what the long-term outcome of patients is, so although the above results suggest that layered strain assessment could be a complementary tool to diagnose patients with CAD, this should be also verified in future experiments.

## 5. Conclusions

In this study, we evaluated the global and local strains of the left ventricle. When we evaluated the global strain of the left ventricle, there was no effect of anatomical variation. The function of three layers of the myocardium was decreased, and all three layers of the myocardium were affected by coronary artery stenosis. The layered strain technique of 2D-STE can evaluate the severity of coronary lesions in patients with NSTE-ACS, and for significant CAD patients, endocardial function is significantly more impaired than epicardial function.

## Figures and Tables

**Table 1:** Comparison of general data.

	Significant CAD group (75 cases)	Nonsignificant CAD group (45cases)	*T* (*χ*^2^)	*P*
Age (years)	62.18 ± 10.73	61.36 ± 10.85	0.43	0.67
Male/female (case)	60/15	35/10	0.08	0.78
Height (cm)	165.10 ± 19.27	169.43 ± 17.18	1.32	0.19
Weight (kg)	69.45 ± 14.16	70.50 ± 10.65	0.46	0.64
Smoking history, *n* (%)	45 (60)	23 (51)	0.91	0.34
Hypertension, *n* (%)	28 (37)	19 (42)	0.28	0.60
Diabetes, *n* (%)	23 (31)	10 (22)	1.00	0.32
Dyslipidemia, *n* (%)	25 (33)	18 (40)	0.54	0.46
Systolic blood pressure (mmHg)	125.58 ± 20.27	129.85 ± 17.29	1.26	0.21
Diastolic blood pressure (mmHg)	74.00 ± 13.51	78.55 ± 14.62	1.83	0.07
Preoperative TnI (ng/mL)	37.5 ± 22.3	30.5 ± 19.0	1.88	0.06
Preoperative BNP (pg/mL)	263.80 ± 464.21	318.54 ± 320.28	0.75	0.45

Note: TnI: troponin I; BNP: B-type natriuretic peptide.

**Table 2:** Comparison of echocardiographic parameters.

Parameters	Significant CAD group (75 cases)	Nonsignificant CAD group (45cases)	*T*	*P*
LVEF	59.1 ± 5.8	60.5 ± 6.2	1.32	0.19
EDV	115.0 ± 22.3	111.0 ± 23.5	0.99	0.32
TLS-endo	−14.6 ± 2.2	−18.9 ± 2.6	0.19	0.000
TLS-mid	−12.8 ± 2.5	−17.5 ± 2.3	0.95	0.000
TLS-epi	−12.0 ± 2.4	−14.6 ± 2.1	6.43	0.000
GLS-endo	−16.0 ± 1.9	−19.3 ± 2.5	8.55	0.000
GLS-mid	−14.5 ± 1.8	−16.5 ± 2.2	5.69	0.000
GLS-epi	−13.2 ± 1.5	−14.2 ± 2.0	3.26	0.001
GCS-endo	−19.1 ± 4.2	−22.3 ± 3.5	4.60	0.000
GCS-mid	−16.2 ± 2.9	−18.9 ± 3.0	6.15	0.000
GCS-epi	−15.1 ± 2.3	−16.8 ± 2.5	4.01	0.001

Note: LVEF: left ventricular ejection fraction; EDV: end-diastolic volume; TLS: territorial longitudinal strain; GLS: global longitudinal strain; GCS: global circumferential strain.

**Table 3:** Comparison of the difference of strain parameters between 1 week after operation and immediately after operation.

	Significant CAD group (75 cases)	Nonsignificant CAD group (45cases)	*T*	*P*
TLS	2.5 ± 2.3	3.9 ± 2.3	3.43	0.000
GLS	2.7 ± 1.8	5.1 ± 2.0	7.16	0.000
GCS	3.3 ± 2.9	6.0 ± 2.5	5.55	0.000

Note: TLS: territorial longitudinal strain; GLS: global longitudinal strain; GCS: global circumferential strain.

**Table 4:** Comparison of ROC curves for each parameter identifying patients with or without significant CAD.

Parameter	Cutoff value	Sensitivity	Specificity	AUC	*P*
LVEF	59.7	0.60	0.58	0.52	0.35
TnI	34.8	0.67	0.55	0.72	0.29
TLSendo	-15.8	0.81	0.86	0.85	0.000
TLSmid	-14.3	0.75	0.82	0.82	0.000
TLSepi	-13.1	0.83	0.59	0.77	0.000
GLSendo	-17.9	0.78	0.79	0.84	0.000
GLSmid	-15.2	0.75	0.82	0.77	0.005
GLSepi	-13.8	0.77	0.75	0.77	0.007
GCSendo	-19.9	0.80	0.79	0.85	0.000
GCSmid	-17.0	0.78	0.76	0.79	0.009
GCSepi	-15.7	0.76	0.73	0.77	0.000

Note: LVEF: left ventricular ejection fraction; TnI: troponin I; TLS: territorial longitudinal strain; GLS: global longitudinal strain; GCS: global circumferential strain.

**Table 5:** Intra- and interobserver variability.

Parameters	Intraobserver variation			Interobserver variation		
	ICC	95% CI	*P* value	ICC	95% CI	*P* value
LVEF	0.92	0.81-0.95	<0.001	0.90	0.78-0.95	<0.001
TnI	0.91	0.80-0.92	<0.001	0.90	0.75-0.92	<0.001
TLSendo	0.93	0.81-0.97	<0.001	0.94	0.84–0.98	<0.001
TLSmid	0.92	0.82-0.97	<0.001	0.93	0.83–0.96	<0.001
TLSepi	0.91	0.82-0.96	<0.001	0.90	0.79–0.97	<0.001
GLSendo	0.92	0.79-0.96	<0.001	0.94	0.79-0.97	<0.001
GLSmid	0.92	0.78-0.95	<0.001	0.93	0.77-0.95	<0.001
GLSepi	0.92	0.75-0.92	<0.001	0.92	0.75-0.93	<0.001
GCSendo	0.92	0.78-0.96	<0.001	0.94	0.78-0.96	<0.001
GCSmid	0.91	0.77-0.94	<0.001	0.93	0.76-0.94	<0.001
GCSepi	0.90	0.76-0.92	<0.001	0.91	0.75-0.93	<0.001

Note: LVEF: left ventricular ejection fraction; TnI: troponin I; TLS: territorial longitudinal strain; GLS: global longitudinal strain; GCS: global circumferential strain.

## Data Availability

All data generated or analyzed during this study are included in this published article.
